# Costs and Benefits of Competitive Traits in Females: Aggression, Maternal Care and Reproductive Success

**DOI:** 10.1371/journal.pone.0077816

**Published:** 2013-10-30

**Authors:** Kristal E. Cain, Ellen D. Ketterson

**Affiliations:** 1 Department of Biology, Indiana University, Bloomington, Indiana, United States of America; 2 Center for the Integrative Study of Animal Behavior, Bloomington, Indiana, United States of America; 3 Research School of Biology, Australian National University, Acton, Australia; Columbia University, United States of America

## Abstract

Recent research has shown that female expression of competitive traits can be advantageous, providing greater access to limited reproductive resources. In males increased competitive trait expression often comes at a cost, e.g. trading off with parental effort. However, it is currently unclear whether, and to what extent, females also face such tradeoffs, whether the costs associated with that tradeoff overwhelm the potential benefits of resource acquisition, and how environmental factors might alter those relationships. To address this gap, we examine the relationships between aggression, maternal effort, offspring quality and reproductive success in a common songbird, the dark-eyed junco (*Junco hyemalis)*, over two breeding seasons. We found that compared to less aggressive females, more aggressive females spent less time brooding nestlings, but fed nestlings more frequently. In the year with better breeding conditions, more aggressive females produced smaller eggs and lighter hatchlings, but in the year with poorer breeding conditions they produced larger eggs and achieved greater nest success. There was no relationship between aggression and nestling mass after hatch day in either year. These findings suggest that though females appear to tradeoff competitive ability with some forms of maternal care, the costs may be less than previously thought. Further, the observed year effects suggest that costs and benefits vary according to environmental variables, which may help to account for variation in the level of trait expression.

## Introduction

Intense competition for limited reproductive resources (mates, territories, etc.) can favor the expression of traits that improve access to these resources, i.e. competitive traits such as ornaments, armaments, or intense same-sex aggression [1]–[5]. However, investment in such traits is often costly. Energy invested in the development or expression of competitive traits is energy no longer available for growth, self-maintenance, or the production and care of offspring. Further, increased trait expression often reduces survival [4], [6]. For males, these costs are generally balanced by improved access to females, leading to increased reproductive success [4]. Our understanding of why females express competitive traits, however, is still limited [1]–[3], [7], [8].

Theory argues that female reproductive success is generally limited by the production and care of offspring, rather than mate number [5], [9]–[11]. Furthermore, because energy invested in the development or expression of competitive traits is no longer available for egg production or offspring care, females should face greater costs than males for competitive trait expression, and experience less benefit [6]. As a consequence, female expression of competitive traits has often been explained as a costly, non-functional by-product of selection on males, reviewed in [1], [3], [12]. However, female reproductive success can also be limited by access to limited, sex-specific reproductive resources (paternal care, nest sites, etc.), rather than solely by ability to produce eggs or care for offspring [1], [3], [8], [12]–[15]. Recent evidence indicates that females also use competitive traits to improve access to resources and that trait expression is often positively related to reproductive success [1], [3], [8], [12]–[14], [16]. Together, these findings suggest that the benefits females accrue from expressing competitive traits may be greater than previously assumed. However, it is currently unclear whether our assumptions about the costs of female trait expression have been similarly inexact.

For species investing in parental care, one of the most important potential costs of competitive trait expression is a negative effect on the amount of time and energy available for offspring production and care [6], [17], [18]. This tradeoff has been well studied in males of many avian species with paternal care [18]–[23]. Because females are often essential caregivers, these costs may be even more substantial for females [6], [10], [14]. Alternatively, because female frequently compete for access to reproductive resources that may have a positive effect on offspring production and care, the relationship between parental care and competitive trait expression in female may be more complex than what is commonly seen in males. However, few studies have directly examined the tradeoff between competitive traits and maternal care [24]. Consequently, it is currently unclear whether females face a similar tradeoff, i.e. a negative relationship between competitive trait expression and investment in offspring. Such data are essential if we are to develop a solid understanding of why females express competitive traits; it is the interplay of costs and benefits that determine the net strength and direction of selection [25].

The dark-eyed junco (*Junco hyemalis*) is a common songbird that is a perennial model for understanding the evolution of morphological, physiological and behavioral traits, e.g. [26]–[30]. Here, we examine the relationship between maternal behavior (brooding and provisioning) and intra-sexual aggression, an important and ubiquitous competitive trait in females that can be costly in terms of time, energy, and risk of injury [8], [14], [16], [31], [32]. We then quantify the fitness consequences of aggression and maternal behavior; directly, by quantifying reproductive success (nest success), and indirectly, by examining egg and nestling mass, proxies for offspring quality [33]. These measures are examined across two breeding seasons that differed in terms of breeding conditions (temperature, precipitation and predation rates). Collectively, these data allow us to examine the potential costs and benefits of competitive trait expression, and how ecology might alter those relationships.

## Methods

### Ethics Statement

This research adhered to the Association for the Study of Animal Behavior/Animal Behavior Society Guidelines for the Use of Animals in Research, the legal requirements of the United States of America (USFWS special use permit number MB093279-2, USGS banding permit number 20261), and The Virginia Department of Game and Fish (#041506). The protocol was approved by the University of Virginia and Indiana University Institutional Animal Care and Use Committees (protocol # 06-242 for both). Research was conducted on the grounds of the Mountain Lake Biological Station, with permission from the station director, in the Jefferson National Forest with permission from The US Department of Agriculture, Forest Service, and on private property with the permission of the landowners. This research did not involve endangered or threatened species.

### Study Species, Site and General Methods

Dark-eyed juncos (*J. h. carolinensis*) are a mildly dimorphic, socially monogamous songbird with biparental care; females alone build the nest, incubate, and brood nestlings, while males assist in feeding and defense [34]. This subspecies of junco are seasonal, partial migrants; males arrive first and establish general use territories (1.316±0.525 ha) that are maintained through the breeding season [34]. Little is known about the role of females in the acquisition and maintenance of territories, however females use non-overlapping home ranges that change in size according to breeding stage (fertile period, 2.44±0.992 ha [35]; nestling period, 0.833 ha±0.156 ha [36]). This study took place on and around Mountain Lake Biological Station, in Giles Co., Virginia (37°22′N, 80°32′W), from April 15–August 10, 2009–2010. Details regarding the study site, species and general practices are available elsewhere [37], [38]. In brief, all resident individuals were captured, banded with serially numbered metal bands and a unique combination of color bands, and aged using a combination of mark-recapture data, and plumage and eye coloration [34]. Once breeding commenced, we searched daily for the nests of all females on the study site. Once a nest was found it was marked, the social pair identified, and the nest monitored daily until egg-laying was complete, then every three days until hatching. As part of a separate experiment, within 24 h of clutch completion, we collected the third-laid egg from each female. If egg order was unknown, e.g. because the nest was found after laying commenced, we selected the largest egg, as the 3rd egg is often largest [34]. Eggs were weighed on a digital scale. After hatching, the nest was monitored every three days until the nest fledged or failed. Nestlings were weighed and measured (nearest 0.1 g) in the afternoon of days 0 (hatch day), 3, 6, and the morning of day 12 (fledging), using the smallest Pesola scale possible (5 g, 10 g or 50 g).

### Aggression Towards an Intruder

Intrasexual aggression was measured in 2009 (*N = *17) and 2010 (*N = *14) by recording behavioral response to a caged conspecific female bird (lure) between days 3–9 of incubation May 15 to June 30; females incubate eggs for 12 days and nests are built throughout the season (May-July). One female was assayed in both years. This behavioral assay we used for intrasexual aggression is described in detail elsewhere [31]. Briefly, during the incubation period we placed a caged same-sex conspecific ∼1 m from the nest and covered the cage with a camouflaged piece of cloth. When the female returned to within 5 m of the nest we removed the cloth and observed the female’s response from ∼15 m using binoculars. We recorded the amount of time spent within 0.25 m, 0.25–1 m, 1–5 m, outside of 5 m, sitting on the nest, and the number of attacks towards the lure (swoops at the lure without contact and actual contacts with the lure’s cage) during a 10 min period.

Females generally responded in one of three ways: attacking persistently throughout the trial, alternating between attacking and sitting on their nest (which was ∼1 m from the intruder), or apparently avoiding interaction by staying >5 m away. Time spent attacking and time spent on the nest were not related (Spearman’s, both years, ρ<0.20, *P*>0.35). To capture this potentially important variation in response style, i.e. differences in tendency to attack, remain in area and occasionally attack, or leave the area entirely, we calculated two distinct aggression scores. We used the amount of time spent within 0.25 m of the lure to gauge overt aggression (time-attacking). Time-attacking was a strong predictor of overtly aggressive behaviors (Spearman’s correlation, time-attacking and dives+hits summed ρ_15_ = 0.8702, *P*<0.0001). We used the total amount of time a female spent within a 1 m radius (time sitting on nest, time attacking and time 0.25–1 m away from nest) to estimate female persistence in the face of a sustained intruder (time-present). Individual females with high time-attacking scores spent most of the trial in direct interaction with the simulated intruder, while those with high time-present scores alternated between attacking and incubating but remained in the immediate area, females that were low in both scores avoided interacting with the intruder. These low scoring females generally left the area and spent the majority of the trial out of sight or more than 5 m away from the simulated intruder. All females eventually returned to incubating, no nests were abandoned due to the trial. To improve normality, variables were square root-transformed. Because there were a number of females that spent zero time within 0.25 m, no transformation could achieve normality; however, linear regressions are generally robust to violations of normality [39]. Date of trial, number of eggs, year, day of incubation, and lure identity had no effect on female response (all *P*>0.30), and all were excluded from further analysis.

### Maternal Behavior

Maternal behavior was estimated by quantifying brooding and provisioning behavior at day 3 post-hatching. Because nest failure is common, sample size was limited (2009, *N* = 13; 2010, *N = *17). Day 3 was chosen because females are still actively warming young, and chicks are large enough to need frequent feedings [34]. The behavioral assay for parental care is described elsewhere [40]. Briefly, we placed a camera 2–4 m from the nest and recorded for 4 h, within 0900–1700. A single observer later analyzed recordings to quantify the number of feeding trips per minute and the length of each brooding bout. For each female we calculated the average length of a brood bout (mean brood bout), excluding the final bout if the female was still on the nest at the end of the observation period. We recorded ambient temperature at 1400 (mid-point for most recordings) using a Campbell CR10 logger located on the study site. There was a negative relationship between average brood length and provisioning rate (per nestling) (*R* ^2^ = 0.297, *F_1, 25_* = 10.14, *P* = 0.0040).

### Statistical Analysis

To examine the relationship between both measures of aggression and maternal behavior, we set the behavioral measure of interest (brooding or provisioning) as the dependent variable and used forward step-wise regression (0.25 to enter, 0.10 to leave) to select informative variables, only final models are reported. The initial full model included age, date of the year, year, ambient temperature, number of nestlings, and measure of aggression. To test for year-specific relationships, we also included a year by aggression interaction term. If the interaction term was significant, we examined relationships independently by year. We used the same approach to examine the relationship between aggression and egg mass; the initial model for egg mass included age, date the egg was collected, measure of aggression, year, and year by aggression interaction. To determine how aggression was related to mean nestling mass at hatch day, day 3, 6, and at fledging we used the same approach; the initial complete model included age, year, date, ambient temperature, number of nestlings, year, and a year by behavior interaction term. Females with more than one nest were included only once, using data from the nesting attempt closest in time to when the behavioral measures were taken. Excluding the one female that was measured in both years had no qualitative effect on the results; all significant effects remained.

**Figure 1:**
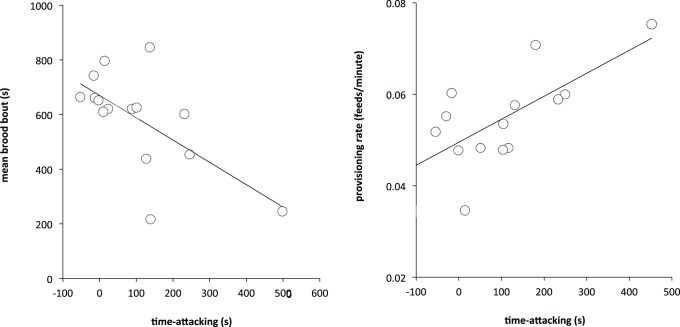
Parental behavior and aggression. Scatter plots relating parental behavior (left: brooding behavior; right: provisioning behavior) to one measure of aggression (time-attacking). Points in the scatter plots are leverage pairs, i.e. the relationship between the variables after controlling for other factors in the model (see Methods and Table 1), akin to partial correlation. Relationships with time-present show similar patterns.

To analyze the effect of aggression on nest fate we coded females as successful if any nesting attempt in a given year produced fledglings, or failed if all attempts were unsuccessful. We then used logistic regression to determine whether behavior was predictive of nest success. For visualization of the relationships in Figure 1, we calculated individual leverage effect pairs from leverage plots. Leverage pairs are derived from the actual residuals from the best-fit line and the residual error without the effect in the model; the result shows the relationships between the two variables after controlling for the other variables in the final model, similar to a partial correlation.

## Results

### Aggression and Maternal Behavior

Measures of aggression toward a same-sex intruder were significantly related to measures of parental behavior, but in opposite directions. Time-attacking and time-present were both negatively related to mean brood bout (Figure 1, Table 1), (time-attacking: final model Adj. *R* ^2^ = 0.54, *F_3, 15_* = 4.39, *P = *0.0291, time-attacking *P = *0.0313), (time-present: final model Adj. *R* ^2^ = 0.40, *F_2, 15_* = 3.91, *P = *0.0183; time-present *P = *0.0299). In contrast, both measures of aggression were positively related to provisioning rate (Figure 1, Table 1), (time-attacking: final model Adj. *R* ^2^ = 0.71, *F_3,14_* = 11.43, *P = *0.0014; time-attacking *P = *0.0159), (time-present: final model Adj. *R* ^2^ = 0.66, *F_3,14_* = 9.31, *P = *0.0030; time-present *P = *0.0103). Year was not a significant predictor of either aggression or parental behavior, nor was there a significant year by behavior interaction (all *P*>0.30).

**Table 1:** Final models of the relationships between measures of parental behavior and measures of aggression.

Measure of maternal effort	Final model results	Trait/Control variable	*b (P)*
mean brood bout	Adj. R^2^ = 0.54	**time-attacking**	**−14.49 (0.031)**
	*F*_3, 15_ = 4.39	date	0.823 (0.089)
	*P* = 0.029	# of nestlings	**−**67.54 (0.249)
provisioning rate	Adj. R^2^ = 0.71	**time-attacking**	**0.0009 (0.016)**
	*F*_3, 14_ = 11.43	date	**−**0.001 (0.0018)
	*P* = 0.001	# of nestlings	0.007 (0.0350)
mean brood bout	Adj. R^2^ = 0.40	**time-present**	**−12.41 (0.030)**
	*F*_2, 15_ = 5.69	date	8.51 (0.072)
	*P* = 0.018		
provisioning rate	Adj. R^2^ = 0.66	**time-present**	**0.001 (0.010)**
	*F*_3, 14_ = 9.31	date	**−**0.002 (0.0001)
	*P* = 0.003	temperature	0.002 (0.010)

Models are multiple regressions. There were no significant year effects, or year by behavior interactions in these models.

### Aggression and Egg Mass

**Figure 2:**
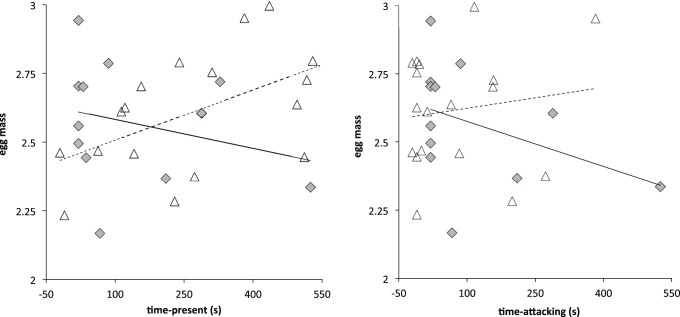
Offspring quality and aggression, by year. Scatter plots illustrating the relationship between aggression measures and one measure of offspring quality (egg mass), according to year. Time-present showed a positive relationship with egg mass in 2009, and a negative, but not significant, relationship in 2010. Conversely, amount of time a female spent attacking was not related to egg mass in 2009 (open triangles and dashed line), but was negatively related to egg mass in 2010 (grey diamonds and solid line). The pattern is similar to the relationships between aggression measures and nestling mass at hatching. Overlapping points are jittered slightly for visual clarity and x-axis begins at **−**50 s to permit viewing of females that did not respond. Raw data presented for visualization; see text and Table 2 for full analysis.

There were no direct relationships between egg mass and aggression (all *P*>0.25); however, there were significant year by aggression interactions. Controlling for the date the egg was collected, in 2009 there was a positive relationship between time-present and egg mass, but in 2010 the relationship was negative (Table 1, Figure 2), (final model Adj. *R* ^2^ = 0.37, *P = *0.0179; time-present *P = *0.21; time-present × year *P* = 0.0043). Examining the relationship between time-present and egg mass separately by year reveals that the relationship was significant in 2009 (*R* ^2^ = 0.33, *P = *0.0131) but not in 2010 (*R* ^2^ = 0.13, *P = *0.2463). There was no statistically detectable relationship between time-attacking and egg mass (*P*>0.40); however, the data followed a similar pattern, no relationship in 2009 and a negative trend in 2010 (Figure 2), (2009: *P*>0.50; 2010: *R* ^2^ = 0.23, *P = *0.1179).

### Aggression, Maternal Behavior and Measures of Reproductive Success

Neither aggression nor mass at hatching differed by year, and when the years were pooled, neither measure of aggression was related to hatchling mass (all *P*>0.25). However, there were significant year by aggression interactions for both measures (time-attacking: final model Adj. *R* ^2^ = 0.24, *P = *0.0168; year by time-attacking interaction *P = *0.0072, *N = *30), (time-present: final model Adj. *R* ^2^ = 0.21, *P = *0.1059; year by time-present interaction *P = *0.0072, *N = *30). Examining the years separately revealed that in 2009 there was no relationship between time-attacking and mass at hatching (Figure 2), (Adj. *R* ^2^ = 0.09, *P = *0.1524, *N = *15); in 2010 there was a pronounced negative relationship between time-attacking and mass at hatching (Figure 2), (Adj. *R^2^* = 0.30, *P = *0.0198, *N = *15). Similarly, there was no relationship between time-present and hatchling mass in 2009 (Adj. *R^2^* = 0.06, *P* = 0.3828, *N = *15), and a negative trend in 2010 (Adj. *R* ^2^ = 0.23, *P = *0.0703, *N = *15). Average nestling mass at hatching was not related to mean brood bout or provisioning rate in either year (P>0.40). Mass on any day after hatching (3, 6 or fledging) was unrelated to time-attacking, time-present, mean brood bout, or provisioning rate (all *P*>0.25).

**Figure 3:**
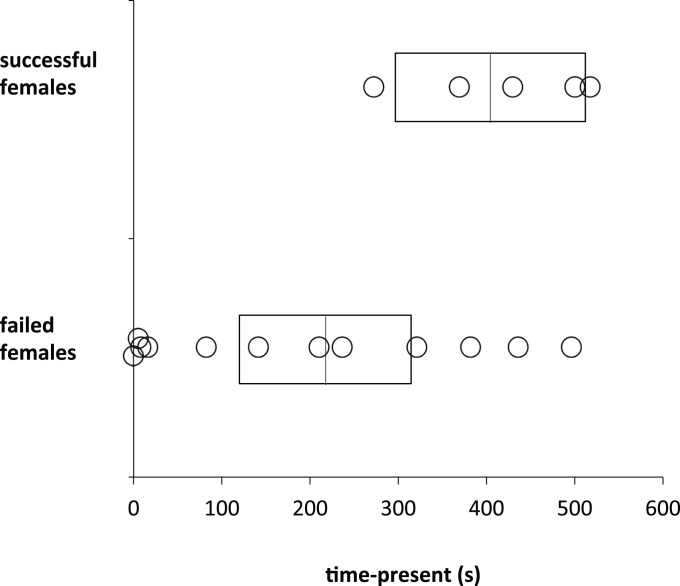
Nest success and aggression in one year. Illustration of aggression score (time-present) in relation to nest fate in 2009. Successful females produced at least one successful nest; failed females had no nest success for the entire season. Time-attacking showed a similar relationship, but was not significant.

In 2009, there were 106 nesting attempts with eggs or young in the entire study population, 26 were successful (∼25%); 5 of 17 focal females produced at least one successful nest (29%), 12 did not, indicating that the focal females were representative. Time-present was positively related to probability of producing a successful nest (Figure 3), (X^2^ *_1,17_* = 6.54, *P = *0.0106); time-attacking was positively, but not significantly, related to nest success (X^2^ *_1,17_* = 1.80, *P* = 0.1806). In 2010, there were 81 nesting attempts with eggs or young, 37 were successful (∼46%); 12 of 14 focal females produced a successful nest (86%). Consequently, we did not have sufficient numbers of unsuccessful females to determine whether there was a relationship between aggression, maternal care and nest success in 2010.

## Discussion

We found that the relationships between aggression and parental behavior were mixed, depending upon the type of parental care (summarized in Table 2). Aggressive females brooded nestlings less, but fed nestlings more frequently. The consequences of being aggressive varied according to year. In one year (2010), egg and hatchling mass were negatively related to aggression, and almost all females had a successful nest, suggesting important costs with no measurable benefit. However, in the other year (2009), aggression was positively related to egg mass, unrelated to hatchling mass, and positively related to nest success. There was no detectable relationship between either measure of aggression and nestling mass at after hatching. This suggests that aggression provides a large but inconsistent benefit.

**Table 2:** Summary of the cost and benefits of competitive trait expression by year.

Measure of aggression	Potential cost or benefit	Direction of relationship	
		2009 (tough year)	2010 (moderate year)
time-attacking	egg mass	0	0
	hatchling mass	0	−
	nest success	+^∧^	n.a.
time-present	egg mass	+	−^∧^
	hatchling mass	0	−^∧^
	nest success	+	n.a.

A plus (+) denotes a positive relationship or a benefit from competitive ability, a minus (−) denotes a negative relationship or cost, a null (0) indicates that no detectable relationship was found. Relationships that were not significant but showed a trend are marked with a caret (^∧^).

### Competitive Ability/Maternal Effort Tradeoff

In support of the idea that females, like males, face tradeoffs between competitive ability and parental care, we found a negative relationship between brooding and both aggression measures. This suggests that females may be limited in their ability to invest in all behaviors and thus face tradeoffs in how they allocate time and effort. In juncos, females alone brood [34] and longer brooding bouts may be more effective at warming developing young, which would allow chicks to devote more energy to growth and less to thermoregulation [33], [40]–[43], suggesting an important potential costs for competitive trait expression. Because the relationships between brooding and aggression have rarely been examined it is difficult to say whether this is a general pattern or an isolated finding.

The relationship between provisioning rate and aggression has been examined more often. In males, competitive traits often function to improve access to mates but often produce a tradeoff, resulting in reduced investment in offspring care [18], [20], [22], [23]. Because females often use competitive traits such as aggression to improve access to resources, rather than to acquire multiple mates, as males are thought to do, competitive ability may improve female ability to invest in offspring. If so, the tradeoff between competitive trait expression and parental care may not always be present [24], and the relationship between aggression and provisioning among females would be less consistent than among males. In support of this possibility, in white-throated sparrow females (*Zonotrichia albicollis*), the more aggressive white-striped morph female provisions less frequently than the less aggressive tan morph female [44], similar to the pattern generally seen in males. However, in tree swallows (*Tachycineta bicolor*), this relationship varies according to population, negative at one study site and a positive trend at the other study site, suggesting that trade-offs may be driven by ecological variables rather than time limitations [45]. Similarly, in female northern cardinals, more exaggerated facemasks are positively associated with both intra-sexual aggression and provisioning rate [46].

We also found that provisioning and brooding were negatively related, which is perhaps not surprising given that females cannot do both simultaneously. Consequently, rather than indicating a cost per se, the negative relationships seen between brooding and aggression, and between brooding and provisioning, may instead be indicative of different behavioral strategies, i.e. some females engaged in a more passive style (low aggression & high brooding) while others engaged in a more active style (high aggression & high provisioning). Finally, it is important to note that male juncos assist in offspring care, and may influence focal female care and nestling growth. Previous experimental work in juncos found positive covariation in male and female provisioning rates, and found that both sexes compensate for reduced care from the mate [47]–[49]. However the nature of the relationship in un-manipulated junco pairs remains to be determined.

### Year Effect on Functional Consequences

The functional consequences of competitive trait expression varied in strength and direction depending on year (see Table 2). This pronounced effect of year on the direction of the relationships suggests that changes in biotic or abiotic variables can alter the costs and benefits associated with competitive phenotypes. The two years differed in weather and predation rates, 2009 was cooler and wetter, with a much higher predation rate relative to 2010. In the tougher year (2009), the relationship between aggression and proxies for offspring quality (egg and nestling mass) were either positive or nonexistent, and aggression was positively related to nest success. In the easier year (2010), aggressive females appeared to pay a cost in terms of smaller eggs and nestlings, and most females experienced some nest success. This suggests that females benefit from a more aggressive behavioral type in tough years, but pay a cost when resources are more abundant. Alternatively, the pronounced year effect may also be attributable to the year differences in predation pressure. Previous work found that aggression towards conspecifics was positively related to aggression towards a simulated predator [31]. Thus, when predation pressure is high, females may benefit if they are better able to deter nest predators. Further research is necessary to determine whether the observed year effects were due to differences in food availability, predation pressure, both, or another variable we are did not measure.

However, regardless of the ecological factor responsible, the annual variation in the functional consequences of aggression reported here adds to the growing body of work suggesting that fluctuating ecological variables can be an important force shaping the strength and direction of selection. For instance, in great tit females (*Parus major*), selection favors fast exploring females in years when food is limited, but slow exploring females when food is more freely available [50]. Similar annual variation has also been reported in Galapagos finches, which experience fluctuating natural selection on beak dimensions [51], and in male lark buntings (*Calamospiza melanocorys*), which experience substantial variation in the annual strength and direction of sexual selection [52].

### Aggression and Egg Mass

In the easier year (2010) we found that aggressive females produced smaller eggs, suggesting either a cost, or that females engage in different parenting strategies. Egg size can have important consequences for developing offspring, suggesting this might be a substantial cost; egg size is positively related to a variety of offspring traits including morphology, survival and growth rate [53]. However, although larger eggs are likely to improve individual offspring survival, investing in offspring quantity, rather than offspring quality, may optimize maternal reproductive success. Larger hatchlings may attract more predators [54] and smaller eggs may permit shorter intra-clutch intervals [55]. This may be important factor for species like the junco, which experience heavy nest predation [31], [55]–[56]. Consequently, caution is warranted when interpreting reduced egg size as a cost, rather than as a strategy.

### Aggression and Nestling Mass

We also found a negative relationship between aggression and hatchling mass in 2009, likely driven by the tendency for more aggressive females to lay smaller eggs that year [53], [57]. We did not measure incubation behavior, but it is likely positively related to brooding, circulating prolactin levels modulate both [58]. If more aggressive females incubate relatively less, as we have shown that they brood less, then nestlings of more aggressive females might also have slower development rates [40]–[43].

However, by 3 days post-hatching, this pattern was no longer detectable, suggesting growth was enhanced in the early nestling period in chicks of aggressive mothers, or suppressed in chicks of less aggressive mothers. The apparent difference in chick growth rates is likely driven by a complex combination of factors, but two possibilities are suggested by other research in this species. First, more aggressive females may have brooded less, but they also provisioned more, and provisioning rate and total food provided are positively correlated in the junco [49]. Thus chicks of aggressive mothers may not be forced to allocate resources to growth *or* thermoregulation, but have sufficient energy for both. A second possibility is that more aggressive females may deposit relatively more testosterone in the yolks of their eggs [57]. More aggressive female juncos produce more testosterone in response to a physiological challenge (injection of gonadotropin releasing hormone or GnRH challenge) [2], and testosterone production ability is positively related to yolk testosterone [59]–[61]. Increased developmental exposure to testosterone can accelerate chick growth and begging [62]–[66]. However, both high levels of testosterone and compensatory growth can have negative long-term consequences [67]–[70], and thus low hatching mass may still be an important cost.

### Aggression and Nest Success

Though maternal care and egg investment can both have important effects on offspring growth and quality, these efforts come to nothing if offspring do not survive. For songbirds, nest success is a crucial component of reproductive success [71]. We found that in one of two years aggressive females had greater nest success, replicating an earlier finding [2]. This suggests that aggressive females experience a major benefit and that there may be strong selection for competitive trait expression in some years.

It is currently unclear why more aggressive female juncos have greater nest success in some years but not others. Females in other species have been shown to compete for limited reproductive resources such as access to nest sites [72], [73], paternal care [32], [74]–[76], mates [77]–[80], territories [81]–[85], dominance [86], [87] or other resources important for reproductive success [88], [89]. For juncos, the main cause of nest failure is predation by small mammals [34], [56], so any female attribute that reduces the probability of predation should be strongly favored. More aggressive females may be better at acquiring a territory with fewer predators or better-protected nest sites, or they may simply be more effective at deterring predators when they approach the nest [2], [31].

Research in other species has also shown that benefits can neutralize the costs of competitive trait expression. For instance, aggressive mothers produce nestlings with lower mass in tree swallows [45], but aggression positively predicts nest site acquisition [73]; aggressive, dominant female baboons (*Papio cynocephalus*) have higher miscarriage rates and reduced fertility, but also experience shorter birth intervals and improved infant survival [90]. Female white-throated sparrows of the white-striped morph are more aggressive and provision less than tan morphs, but experience similar overall fitness [20]. In dung beetles, females with large horns for their body size had higher reproductive success when resources were limited [89], and showed no detectable fecundity cost [91], suggesting that the level of investment in competitive traits may be due to female quality rather than a tradeoff.

Overall, our finding of relatively minor costs, coupled with major benefits, suggests that our understanding to date of what makes a ‘good’ mother may be too simplistic. If we had measured only brooding behavior, we might have been inclined to label low brooding females poor quality mothers; however our findings suggest that these females are high quality mothers in other measures, i.e. provide more food or increased chance of survival. In fact, one might hypothesize that less aggressive, less competitive females have reduced access to important limited resources, but offset this loss by investing more in maternal care (spend more time brooding or make larger eggs).

Finally, there are many other potential costs and benefits that this study did not measure, e.g. survival, offspring recruitment, attractiveness to males. Female expression of competitive traits may increase access to important resources in the non-breeding season [3], [79], [88], [92]–[96], which could strengthen the observed benefits of aggression. However, competitive traits can also have negative effects on survival [8], [14], [16]. Further, while female-female fights may be less frequent, they are also appear to be less ritualized and more likely to result in injury or death, possibly due to differences in the payoff of success and costs of failure [97], [98]. Consequently, a more complete estimate of fitness is necessary before we can conclude whether, and to what extent, social selection is favoring the expression of competitive traits in females (Cain & Rosvall unpublished) [3], [96].

## Conclusions

Our findings suggest that although females pay a cost for expressing competitive traits, here intrasexual aggression, these costs may be outweighed by benefits, at least in some years. These results add to the growing body of work from a wide-variety of taxa supporting the hypothesis that female competitive traits often function in manner analogous to male competitive traits, i.e. they improve reproductive success via access to limited resources, whether those resources are mates, or some other reproductive resources (Cain & Rosvall unpublished) [1]–[3], [8], [13]–[16], [91].
